# Association Between Therapeutic Interventions and Sleep Disorders in Patients with Breast Cancer: A National Population-Based Cohort Study

**DOI:** 10.3390/cancers18030397

**Published:** 2026-01-27

**Authors:** Dooreh Kim, Hye Sun Lee, Soyoung Jeon, Jinah Lee, Woo-Chan Park, Jooyoung Oh, Chang Ik Yoon

**Affiliations:** 1 Division of Breast Surgery, Department of Surgery, Seoul St Mary’s Hospital, College of Medicine, The Catholic University of Korea, Seoul 06591, Republic of Korea; rlaenfpd@gmail.com (D.K.); jinahsmile@catholic.ac.kr (J.L.); wcpark@catholic.ac.kr (W.-C.P.); 2 Biostatistics Collaboration Unit, Yonsei University College of Medicine, Seoul 03722, Republic of Korea; hslee1@yuhs.ac (H.S.L.); jsy0331@yuhs.ac (S.J.); 3 Department of Psychiatry, Gangnam Severance Hospital, Yonsei University College of Medicine, Seoul 06273, Republic of Korea; 4 Institute of Behavioral Sciences in Medicine, Yonsei University College of Medicine, Seoul 06273, Republic of Korea

**Keywords:** breast cancer, insomnia, sleep disorder, chemotherapy, endocrine therapy

## Abstract

**Background**: Breast cancer survivors often experience long-term endocrine- and chemotherapy-related side effects, including sleep disorders, anxiety, and depression. Sleep disorders are particularly prevalent and affect patient adherence and quality of life. This study examined the prevalence and risk factors for sleep disorders in patients with breast cancer based on treatment exposure. **Methods**: Patients with breast cancer (2009–2015) were identified from the Health Insurance Review and Assessment database. They were categorized by chemotherapy exposure and further by endocrine or taxane use. Sleep disorders, anxiety, and depression were assessed using diagnostic and prescription codes. Propensity score matching and Cox proportional hazard models were applied to adjust confounders and evaluate risk factors. **Results**: Among the 62,714 patients, those receiving endocrine therapy had a higher risk of sleep disorders (hazard ratio [HR], 1.276; 95% confidence interval [CI], 1.087–1.497; *p* = 0.003), irrespective of tamoxifen or aromatase inhibitor use. In chemotherapy-treated patients, taxane exposure significantly increased sleep disorder risk (HR, 1.268; 95% CI, 1.159–1.389; *p* < 0.001). The cumulative incidence of sleep disorders peaked within two years post treatment and remained elevated over time. Anxiety and depression rates did not differ significantly between the treatment groups. **Conclusions**: Endocrine therapy and taxane chemotherapy are independent risk factors for sleep disorders in patients with breast cancer. Screening and interventions are essential for improving long-term well-being. Future studies should explore personalized approaches for managing treatment-related sleep disturbance.

## Simple Summary

This population-based study of 62,714 breast cancer patients identified from the Korean Health Insurance Review and Assessment database revealed that endocrine therapy and taxane chemotherapy independently increased the risk of sleep disorders, while anxiety and depression rates did not differ between treatment groups. Sleep disorders were most frequent within two years after treatment and remained elevated thereafter, highlighting the need for early screening and tailored interventions to improve long-term quality of life among breast cancer survivors.

## 1. Introduction

Breast cancer often develops in the early decades of life and generally has a better prognosis than other types of cancer do, owing to advances in diagnosis and treatment. Nonetheless, breast cancer survivors face long-term iatrogenic effects of endocrine treatment, including fatigue, chronic pain, and vasomotor symptoms, all of which may negatively affect quality of life and mental health throughout their lifetime [1]. Despite these known iatrogenic effects, the specific longitudinal association between various therapeutic approaches and the risk of developing clinical mental illness remains insufficiently explored. The majority of breast cancer cases occur in female patients, and previous studies indicate that these patients experience more profound psychosocial distress, including anxiety and depression [2].

Sleep disorders, particularly insomnia, are highly prevalent in cancer. Research indicates that up to 95% of these individuals experience sleep disturbances after their diagnosis [3]. The diverse therapeutic modalities for breast cancer entail side effects that can precipitate sleep disturbances in addition to the psychological impact of the diagnosis itself. Moreover, mental illness can affect treatment adherence, leading to suboptimal outcomes and increased risk of recurrence [4].

Endocrine treatment for breast cancer often exacerbates menopausal symptoms, with hot flashes being the most common, frequently leading to sleep disturbances [5]. Mood disorders related to endocrine therapy, such as anxiety and depression, can also trigger new-onset sleep disturbances, and these issues are closely interconnected. These symptoms are not limited to young premenopausal patients with breast cancer. According to a substudy of the Tamoxifen Exemestane Adjuvant Multinational trial, levels of sleep disturbance were higher in patients receiving aromatase inhibitors than in those on tamoxifen [6]. In peri- and postmenopausal women, reduced estradiol levels disrupt circadian rhythms, causing increased nocturnal arousal [7].

Factors that contribute to diminished mental health and reduced quality of life include the experience of undergoing chemotherapy. A diagnosis of advanced-stage cancer requiring chemotherapy can itself be a source of psychological distress [8] and long-term adverse events such as fatigue may further exacerbate the vulnerability of this population. Taxanes, frequently administered chemotherapeutic agents for breast cancer, affect both the central and peripheral nervous systems. Notably, chemotherapy-induced peripheral neuropathy (CIPN) occurs in approximately 80–97% of patients treated with taxane chemotherapy, with many cases becoming chronic [9,10]. The persistent nature and severity of CIPN symptoms contribute to substantial psychological distress [11].

This study aimed to investigate the prevalence and risk factors for sleep disorders, anxiety, and depression in patients with breast cancer using a nationwide database in Korea. The patients were stratified into two cohorts based on their treatment exposure. This study aimed to identify which group of patients is more affected by debilitating sleep disorders, enabling targeted monitoring and intervention. Additionally, we sought to determine whether sleep disturbances occur alongside mood symptoms, such as anxiety and depression, or whether they arise independently as adverse effects of specific cancer treatments.

## 2. Materials and Methods

### 2.1. Study Design

**Figure 1:**
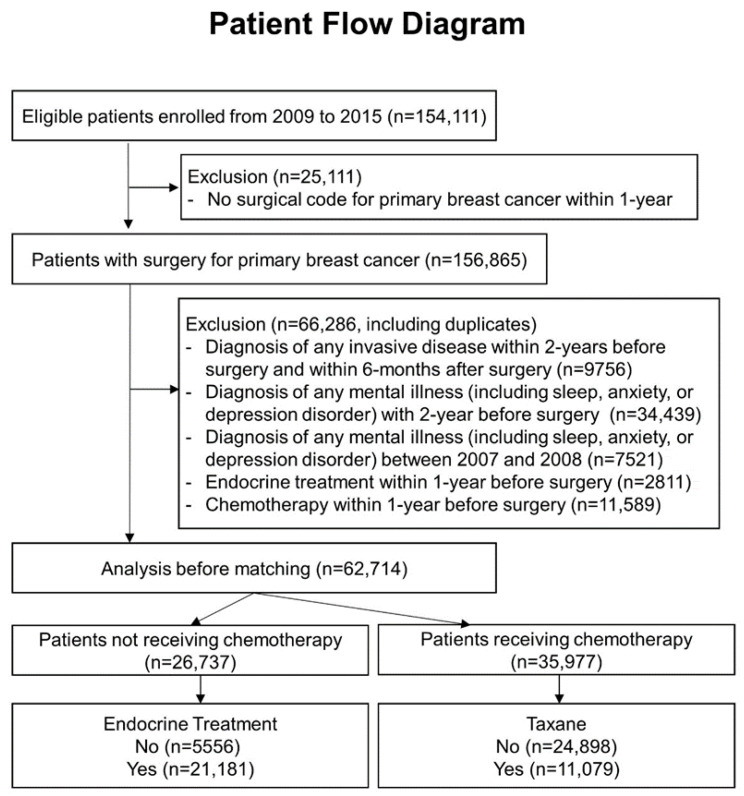
Flow diagram of patients enrolled in the retrospective cohort study.

Individuals diagnosed with in situ or invasive breast cancer between 2009 and 2015 were identified using the Health Insurance Review and Assessment Claims database. Patients without a surgical code within one year of diagnosis were excluded because they were considered to have a systemic disease. A two-year wash-out period was applied to ensure no prior diagnosis of breast cancer or any other cancer (Supplementary Table S1). Patients with a history of mental illness, including sleep, anxiety, or depressive disorders, were also excluded. To exclude those who underwent primary systemic therapy or palliative treatment, any treatment received within 1 year prior to surgery was excluded. The treatment modalities included endocrine therapy, chemotherapy, and targeted therapy, which were determined using prescription codes (Supplementary Table S2). Initially, 62,714 patients were eligible for analysis. These patients were divided into two cohorts based on whether they received chemotherapy, as chemotherapy can influence the development of sleep disorders. Each subgroup was further subdivided based on endocrine therapy and taxane exposure (Figure 1).

### 2.2. Assessment of Mental Illness

The primary objective was to determine the incidence of mental illness in patients with breast cancer according to treatment modality. Mental illness was broadly classified into three main categories—anxiety, depression, and sleep disorders—with detailed diagnoses available in Supplementary Table S3. Moreover, mental illness was operationally defined using two criteria based on medical claims data. Under Definition 1, a patient was identified as having a mental illness if the relevant diagnostic code was recorded on a claim. To increase diagnostic specificity, Definition 2 required the presence of both the diagnostic code and a record of medication prescribed for the specific disorder on the same claim. Sleep disorders constitute a highly heterogeneous disease entity. Therefore, they are defined only when the use of concomitant sleep medication is documented. Given that benzodiazepines are commonly prescribed not only for sleep but also for concomitant anxiety, and that most antidepressants, such as selective serotonin reuptake inhibitors and serotonin–norepinephrine reuptake inhibitors, primarily target depressive and anxiety symptoms, we defined sleep disorder only when medications primarily intended for sleep induction were prescribed (Supplementary Table S2). Comorbidity data were also collected and calculated using the Charlson Comorbidity Index (CCI) score (Supplementary Table S4) to account for potential confounding variables. These comorbidities were identified using diagnostic codes within two years preceding the enrollment date.

### 2.3. Statistical Analysis

Statistical analyses were conducted to compare the baseline demographic and clinical characteristics between the two study groups using t-tests and chi-square tests. The cumulative incidence rates of mental illness in both groups were depicted using Kaplan–Meier curves and compared using the log-rank test. Cox proportional hazard models were used to calculate hazard ratios (HRs) and the corresponding 95% confidence intervals (CIs) to investigate the occurrence of mental illness, adjusting for confounding variables. Statistical significance was set at a two-sided *p*-value of less than 0.05.

Randomization was performed using an algorithm in SAS software (v9.4; SAS Institute, Cary, NC, USA). To mitigate bias, propensity scores were estimated and used to match the treatment populations. Each patient’s score was computed using logistic regression analysis, factoring in variables such as age, type of adjuvant treatment, and comorbidities. Patients were matched based on propensity scores using a nearest-neighbor greedy algorithm. Furthermore, a 1:1 propensity score matching technique was implemented to optimize the patient count for breast cancer while minimizing potential biases in the estimated outcomes.

## 3. Results

### 3.1. Patient Characteristics

The patient cohorts were categorized into two groups based on whether they had received chemotherapy. The initial cohort comprised 26,737 patients with breast cancer who did not undergo chemotherapy. These patients were compared in terms of their receipt of endocrine therapy before and after propensity score matching (Table 1). Before matching, significant differences in age distribution, sex, radiation therapy, and sleep disorders were observed between groups. Patients receiving endocrine therapy tended to be younger, with the highest proportion in their 40s. Furthermore, patients in the endocrine therapy group had a higher tendency to receive RT. These differences were mitigated after matching. However, even after matching the clinical variables, the incidence of sleep disorders remained higher in the group that received endocrine therapy. The choice of endocrine therapy agent (tamoxifen or aromatase inhibitor) did not influence the incidence of sleep disorders (Supplementary Table S5). There were no significant differences in the prevalence of anxiety or depression disorders between the two groups before or after matching.

In the second cohort, the clinical characteristics of breast cancer patients receiving chemotherapy were compared based on taxane use (Table 2). Prior to propensity score matching, patients who underwent taxane-based chemotherapy were more likely to receive endocrine and radiation therapy, suggesting an advanced stage; however, this difference was no longer significant after matching. Interestingly, a slight but statistically significant difference was observed only after matching, with a higher proportion of males receiving taxanes (*p* = 0.035). Patients treated with taxanes exhibited a significantly higher prevalence of sleep disorders, as defined by both Definitions 1 and 2, before matching, and these differences persisted after matching. Again, there were no significant differences in the prevalence of anxiety or depression disorders between the groups.

### 3.2. Cumulative Incidence of Sleep Disorder

**Figure 2:**
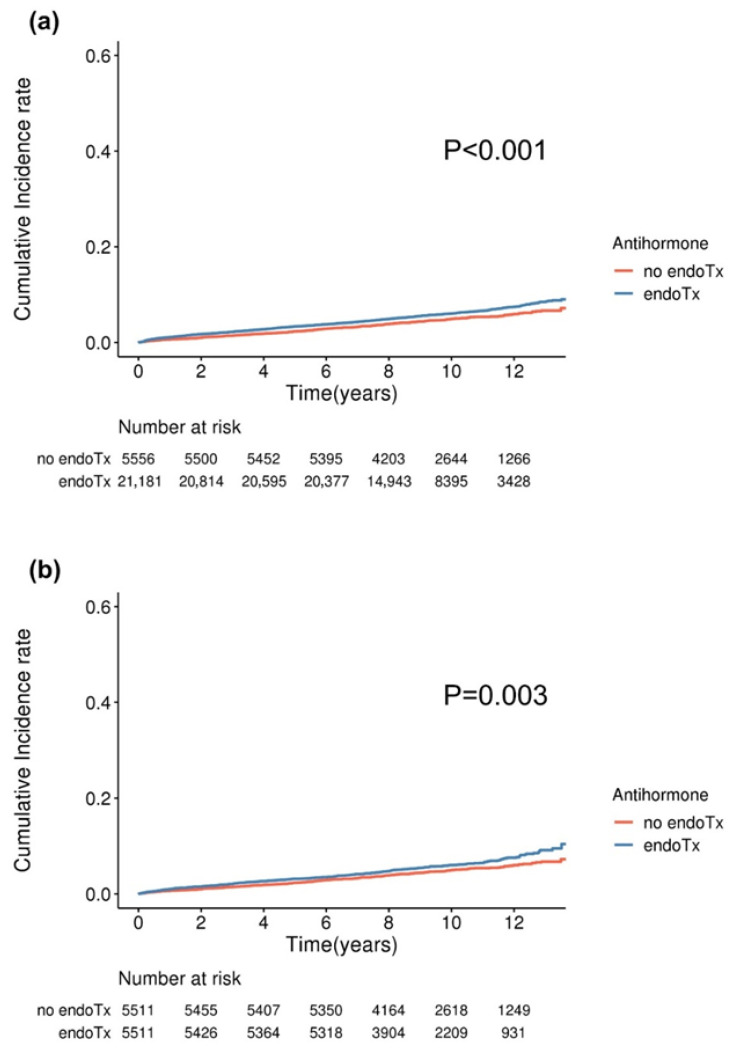
Kaplan–Meier analysis of the incidence of sleep disorder according to endocrine treatment in breast cancer patients not receiving chemotherapy. (**a**) Cumulative incidence before matching (median follow-up, 9.37 ± 2.42 years); (**b**) cumulative incidence after matching (median follow-up, 9.54 ± 2.41 years).

**Figure 3:**
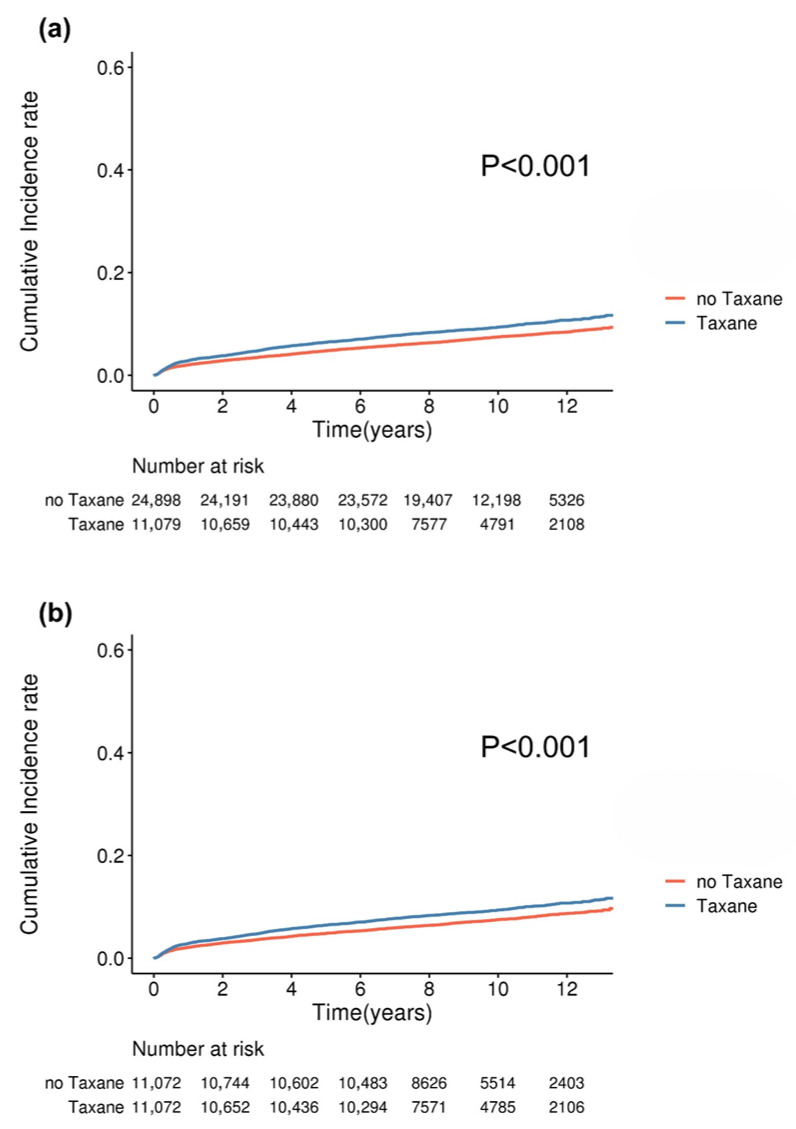
Kaplan–Meier analysis of the incidence of sleep disorder according to taxane use in breast cancer patients receiving chemotherapy. (**a**) Cumulative incidence of sleep disorder before matching (median follow-up, 9.80 ± 2.73 years); (**b**) cumulative incidence of sleep disorder after matching (median follow-up, 10.06 ± 2.01 years).

The cumulative incidence of sleep disorders gradually increased in the subgroups across each cohort before and after matching. This divergence remained consistent throughout the extended follow-up period, with a median duration of 9.2 years. Notably, patients undergoing endocrine therapy demonstrated a significantly distinct cumulative incidence of sleep disorders compared with those who did not receive such treatments (Figure 2). Regardless of whether patients received tamoxifen or an aromatase inhibitor, the cumulative incidence of sleep disorders was not significantly different between the endocrine regimens (Supplementary Figure S1). Similarly, individuals treated with taxane-based chemotherapy exhibited a markedly different cumulative incidence of sleep disorders than those treated with non-taxane-based regimens (Figure 3). Further evaluation presented the annual hazard rate of sleep disorder incidence in a graphical format, revealing that the incidence of sleep disorders peaked within the first 1–2 years (Supplementary Figure S2). Moreover, the graph shows that endocrine therapy exerts a more prolonged influence on the incidence of sleep disorders than chemotherapy.

### 3.3. Risk of Developing Sleep Disorders

The risk of developing sleep disorders in patients with breast cancer was analyzed using Cox proportional hazard models in both cohorts, as shown in Table 3 and Table 4, respectively. In the non-chemotherapy cohort, age was a significant factor in both the univariate and multivariate analyses, both before and after matching. The CCI scores showed a significant association in the univariate analysis but not in the multivariate analysis after matching. Endocrine and radiation therapies were associated with a higher risk of sleep disorders in all analyses. After matching, in the multivariate analysis, patients who received endocrine therapy were 27% more likely to develop sleep disorders (HR, 1.276; 95% CI, 1.087–1.497; *p* = 0.003), and patients who received radiation therapy were 44% more likely to develop sleep disorders (HR, 1.441; 95% CI, 1.229–1.689; *p* < 0.001). Furthermore, when patients were subdivided based on the type of endocrine therapy (tamoxifen or an aromatase inhibitor), the specific antihormonal agent used was not associated with the incidence of sleep disorders (Supplementary Table S6).

In the chemotherapy cohort, variables such as age, sex, and CCI were not significantly associated with the development of sleep disorders before and after matching. Although age showed a borderline significant association in the multivariate analysis before matching (HR, 1.039; 95% CI, 1.000–1.079; *p* = 0.048), it was not significant after matching. All three treatment modalities, including the type of chemotherapy, endocrine therapy, and radiation therapy, demonstrated a substantial association with the incidence of sleep disorders prior to matching. However, after matching, radiation therapy was no longer a significant factor. Patients undergoing endocrine therapy were more likely to develop sleep disorders in both univariate and multivariate analyses both before and after matching (HR, 1.171; 95% CI, 1.051–1.305; *p* = 0.004). The risk of developing sleep disorders was particularly pronounced in patients receiving taxane-based chemotherapy, with an HR of 1.268 (95% CI, 1.159–1.389; *p* < 0.001) in the multivariate analysis after matching.

## 4. Discussion

This large-scale nationwide cohort study investigated the association between specific breast cancer treatments and the development of sleep disorders, anxiety, and depression. Contrary to the common assumption that sleep disturbances in patients with cancer are secondary to anxiety or depressive symptoms [12] and given that insomnia is a frequent manifestation of both conditions, our findings suggest that sleep disorders can emerge independently as distinct consequences of endocrine therapy and chemotherapy.

The observed increase in sleep disorder incidence across treatment modalities carries meaningful clinical implications. First, sleep disturbance is a major determinant of health-related quality of life in breast cancer survivors, contributing to fatigue, cognitive complaints, and psychological distress, all of which may undermine treatment adherence and overall well-being. Second, accumulating evidence suggests that persistent sleep dysregulation may promote chronic systemic inflammation and activation of stress pathways, which have been associated with worse cancer-related outcomes [13,14]. Given that both endocrine therapy and systemic chemotherapy are commonly administered to young patients who are otherwise expected to have long survivorship, early identification and proactive management of sleep problems represent an important dimension of comprehensive supportive care.

By analyzing two separate cohorts based on chemotherapy exposure and applying rigorous propensity score matching, we found that both endocrine therapy and taxane-based chemotherapy independently increased the risk of sleep disorders, with no corresponding increase in the incidence of anxiety or depression. These findings underscore the need for treatment-specific monitoring and supportive interventions, particularly during the early post-treatment period when the incidence of sleep disturbances peaks, as observed in our study.

Our findings reinforce existing evidence that endocrine therapy is a major contributor to sleep disturbances in breast cancer patients [15]. Previous studies have linked aromatase inhibitors and tamoxifen to vasomotor symptoms such as hot flashes [16,17], which interfere with sleep. Moreover, fluctuations in estrogen levels during endocrine therapy may disrupt circadian rhythms and promote nocturnal arousal, especially in peri- and postmenopausal women [18]. However, our study extends this knowledge using national population cohort data by demonstrating that the increased risk of sleep disorder is present regardless of the specific endocrine agent used. Unlike many previous studies that relied on patient-reported outcomes [19,20], we defined sleep disorders using objectively recorded prescription data to enhance the reliability of the findings. The lack of a corresponding increase in anxiety or depression further supports the hypothesis that sleep disturbance may occur as a distinct treatment-related condition rather than as a secondary symptom of emotional distress.

Another important contribution of our study is the identification of a significantly increased risk of sleep disorders in patients treated with taxane-based chemotherapy. Taxanes are known to induce CIPN [21], a chronic and often debilitating condition that affects up to 97% of treated patients. Although we could not directly assess CIPN in this dataset, the persistent difference in the prevalence of sleep disorders between taxane and non-taxane regimens suggests that a neurotoxic mechanism may be involved. Sensory disturbances, pain, and functional impairments associated with CIPN can disrupt sleep continuity and contribute to prolonged sleep difficulties. These findings emphasize the need to consider the long-term neuropsychiatric sequelae of taxane exposure in survivorship care planning.

In addition to chemotherapy-induced peripheral neuropathy, emerging evidence suggests that taxanes can disrupt sleep regulation through neuroinflammatory and neuroendocrine pathways. Taxane-based chemotherapy has been shown to upregulate pro-inflammatory cytokines such as IL-6 and TNF-α, which are known to interfere with hypothalamic sleep–wake regulation and promote fragmented sleep patterns [22]. Paclitaxel and docetaxel have also been associated with activation of the hypothalamic–pituitary–adrenal system and alterations in cortisol rhythms, which contribute to insomnia and circadian disruption.

Our study has several limitations. First, although we employed a prescription-based definition to objectively identify sleep disorders, we could not directly assess the subjective severity, sleep quality, or associated psychological distress. Second, our case definition required documentation of medications primarily prescribed for sleep induction and maintenance, which may have led to an underestimation of the true prevalence by excluding non-pharmacologically managed or subclinical cases. Third, we did not evaluate the qualitative aspects of treatment exposure, such as dosage, duration, or adherence to endocrine or chemotherapy regimens, which may have modulated the observed associations. Despite these limitations, the strengths of this study include its large, nationally representative cohort, robust methodological design, and long-term follow-up, which enhanced the validity and clinical relevance of our findings.

## 5. Conclusions

Endocrine therapy and taxane chemotherapy are independent predictors of sleep disturbances in breast cancer patients. Routine screening and targeted interventions are essential to improve long-term outcomes. Future studies should focus on personalized strategies to manage treatment-related sleep disorders.

## Figures and Tables

**Table 1:** Comparison of clinical characteristics according to endocrine treatment in breast cancer patients not receiving chemotherapy.

	Before Matching			After Matching		
	Not Receiving Endocrine Therapy, n = 5556 (%)	Receiving Endocrine Therapy, n = 21,181 (%)	*p* Value	Not Receiving Endocrine Therapy, n = 5511 (%)	Receiving Endocrine Therapy, n = 5511 (%)	*p* Value
Age (year)			<0.001			>0.999
20–29	120 (2.16)	222 (1.05)		80 (1.45)	80 (1.45)	
30–39	779 (14.0)	2461 (11.6)		779 (14.1)	779(14.1)	
40–49	1858 (33.4)	9520 (45.0)		1858 (33.7)	1859 (33.7)	
50–59	1705 (30.7)	5330 (25.2)		1705 (30.9)	1708 (31)	
60–69	611 (11.0)	2316 (10.9)		611 (11.1)	611 (11.1)	
70–79	362 (6.5)	1157 (5.5)		362 (6.6)	362 (6.6)	
80–	121 (2.2)	175 (0.8)		116 (2.1)	112 (2.0)	
Gender			0.0304			0.6828
Male	14 (0.3)	98 (0.5)		13 (0.2)	11 (0.2)	
Female	5542 (99.7)	21,083 (99.5)		5498 (99.8)	5500 (99.8)	
CCI (Weight number, mean ± SD)	3.452 ± 1.896	3.452 ± 1.831	0.9933	3.457 ± 1.899	3.434 ± 1.842	0.5179
Radiation			<0.001			0.9845
Not done	3308 (59.5)	5797 (27.4)		3263 (59.2)	3262 (59.2)	
Done	2248 (40.5)	15,384 (72.6)		2248 (40.8)	2249 (40.8)	
Sleep disorder (Definition 1)			0.0363			0.399
No	5127 (92.3)	19,360 (91.4)		5083 (92.2)	5059 (91.8)	
Yes	429 (7.7)	1821 (8.6)		428 (7.8)	452 (8.2)	
Sleep disorder (Definition 2)			0.0024			0.0139
No	5281 (95.1)	19,906 (94.0)		5236 (95.0)	5177 (94.0)	
Yes	275 (5.0)	1275 (6.0)		275 (5.0)	334 (6.0)	
Anxiety disorder (Definition 1)			0.9069			0.694
No	4945 (89.0)	18,840 (89.0)		4903 (89.0)	4890 (88.7)	
Yes	611 (11.0)	2341 (11.0)		608 (11.0)	621 (11.3)	
Anxiety disorder (Definition 2)			0.7985			>0.999
No	5066 (91.2)	19,336 (91.3)		5022 (91.1)	5022 (91.1)	
Yes	490 (8.8)	1845 (8.7)		489 (8.9)	489 (8.9)	
Depression disorder (Definition 1)			0.3752			0.459
No	4977 (89.58)	18,886 (89.16)		4935 (89.55)	4911 (89.11)	
Yes	579 (10.42)	2295 (10.84)		576 (10.45)	600 (10.89)	
Depression disorder (Definition 2)			0.671			0.7424
No	5048 (90.86)	19,205 (90.67)		5006 (90.84)	4996 (90.66)	
Yes	508 (9.14)	1976 (9.33)		505 (9.16)	515 (9.34)	

CCI, Charlson Comorbidity Index; SD, standard deviation. Definition 1: If the diagnosis code is confirmed on the claim, it is operationally defined as having been diagnosed with the disease. Definition 2: If both the diagnosis code and medication for disease are identified on the claim, the patient is operationally defined as having been diagnosed with the disease.

**Table 2:** Comparison of clinical characteristics according to taxane use in breast cancer patients receiving chemotherapy.

	Before Matching			After Matching		
	Not Receiving Taxane, n = 24,898 (%)	Receiving Taxane, n = 11,079 (%)	*p* Value	Not Receiving Taxane, n = 11,072 (%)	Receiving Taxane, n = 11,072 (%)	*p* Value
Age (year)			<0.001			>0.999
20–29	351 (1.41)	177 (1.60)		177 (1.60)	177 (1.60)	
30–39	3500 (14.06)	1912 (17.26)		1908 (17.23)	1908 (17.23)	
40–49	10,507 (42.20)	4774 (43.09)		4777 (43.14)	4773 (43.11)	
50–59	7331 (29.44)	3135 (28.30)		3138 (28.34)	3133 (28.30)	
60–69	2621 (10.53)	937 (8.46)		930 (8.40)	937 (8.46)	
70–79	572 (2.30)	141 (1.27)		140 (1.26)	141 (1.27)	
80–	16 (0.06)	3 (0.03)		2 (0.02)	3 (0.03)	
Gender			0.9217			0.0354
Male	86 (0.35)	39 (0.35)		21 (0.19)	37 (0.33)	
Female	24,812 (99.65)	11,040 (99.65)		11,051 (99.81)	11,035 (99.67)	
CCI (Weight number, mean ± SD)	3.673 ± 2.203	3.838 ± 2.388	<0.0001	3.832 ± 2.376	3.831 ± 2.375	0.9865
Radiation			<0.0001			0.8842
Not done	5660 (22.73)	1806 (16.30)		1798 (16.24)	1806 (16.31)	
Done	19,238 (77.27)	9273 (83.70)		9274 (83.76)	9266 (83.69)	
Sleep disorder (Definition 1)			<0.0001			<0.0001
No	22,288 (89.52)	9682 (87.39)		9880 (89.23)	9676 (87.39)	
Yes	2610 (10.48)	1397 (12.61)		1192 (10.77)	1396 (12.61)	
Sleep disorder (Definition 2)			<0.0001			<0.0001
No	23,016 (92.44)	10,033 (90.56)		10,218 (92.29)	10,027 (90.56)	
Yes	1882 (7.56)	1046 (9.44)		854 (7.71)	1045 (9.44)	
Anxiety disorder (Definition 1)			0.1491			0.1015
No	20,903 (83.95)	9368 (84.56)		9273 (83.75)	9362 (84.56)	
Yes	3995 (16.05)	1711 (15.44)		1799 (16.25)	1710 (15.44)	
Anxiety disorder (Definition 2)			0.0917			0.0669
No	21,822 (87.65)	9780 (88.28)		9685 (87.47)	9774 (88.28)	
Yes	3076 (12.35)	1299 (11.72)		1387 (12.53)	1298 (11.72)	
Depression disorder (Definition 1)			0.2386			0.4535
No	21,694 (87.13)	9703 (87.58)		9660 (87.25)	9697 (87.58)	
Yes	3204 (12.87)	1376 (12.42)		1412 (12.75)	1375 (12.42)	
Depression disorder (Definition 2)			0.1451			0.2872
No	22,027 (88.47)	9860 (89.00)		9804 (88.55)	9854 (89.00)	
Yes	2871 (11.53)	1219 (11.00)		1268 (11.45)	1218 (11.00)	

CCI, Charlson Comorbidity Index; SD, standard deviation. Definition 1: If the diagnosis code is confirmed on the claim, it is operationally defined as having been diagnosed with the disease. Definition 2: If both the diagnosis code and medication for disease are identified on the claim, the patient is operationally defined as having been diagnosed with the disease.

**Table 3:** Risk of developing sleep disorder in breast cancer patients not receiving chemotherapy from analyses using the Cox proportional hazard models (Definition 2).

	Before Matching		Before Matching		After Matching		After Matching	
	Univariate Analysis		Multivariate Analysis		Univariate Analysis		Multivariate Analysis	
	HR (95% CIs)	*p* Value	HR (95% CIs)	*p* Value	HR (95% CIs)	*p* Value	HR (95% CIs)	*p* Value
Age (per 10-year)	1.122 (1.076–1.171)	<0.001	1.130 (1.081–1.182)	<0.001	1.174 (1.104–1.250)	<0.0001	1.172 (1.099–1.251)	<0.0001
Gender		0.280		0.2583		0.4653		0.403
Male	Reference		Reference		Ref		Ref	
Female	1.718 (0.644–4.583)		1.764 (0.629–4.719)		2.809 (0.176–44.943)		3.266 (0.204–52.291)	
CCI	1.040 (1.014–1.066)	0.0019	1.025 (0.999–1.051)	0.0627	1.054 (1.014–1.095)	0.0072	1.031 (0.992–1.073)	0.1243
Endocrine therapy		0.0002		0.0077		0.0027		0.0028
Not done	Reference		Reference		Ref		Ref	
Done	1.280 (1.124–1.459)		1.201 (1.050–1.375)		1.276 (1.088–1.498)		1.276 (1.087–1.497)	
Radiation		<0.0001		<0.0001		<0.0001		<0.0001
Not done	Reference		Reference		Ref		Ref	
Done	1.299 (1.164–1.450)		1.283 (1.144–1.439)		1.432 (1.222–1.679)		1.441 (1.229–1.689)	

**Table 4:** Risk of developing sleep disorder in breast cancer patients receiving chemotherapy from analyses using the Cox proportional hazard models (Definition 2).

	Before Matching		Before Matching		After Matching		After Matching	
	Univariate Analysis		Multivariate Analysis		Univariate Analysis		Multivariate Analysis	
	HR (95% CIs)	*p* Value	HR (95% CIs)	*p* Value	HR (95% CIs)	*p* Value	HR (95% CIs)	*p* Value
Age (per 10-year)	1.024 (0.986–1.062)	0.216	1.039 (1.000–1.079)	0.048	1.009 (0.962–1.058)	0.720	1.014 (0.966–1.064)	0.579
Gender		0.1765		0.1567		0.1834		0.1565
Male	ref		ref		ref		ref	
Female	1.737 (0.780–3.870)		1.786 (0.800–3.984)		2.562 (0.641–10.243)		2.726 (0.681–10.913)	
CCI	1.004 (0.988–1.020)	0.617	1.001 (0.985–1.017)	0.8909	1.003 (0.984–1.022)	0.7571	1.004 (0.985–1.023)	0.7026
Chemotherapy		<0.0001		<0.0001		<0.0001		<0.0001
Excluding taxane	ref		ref		ref		ref	
Including taxane	1.293 (1.198–1.394)		1.281 (1.187–1.382)		1.267 (1.157–1.387)		1.268 (1.159–1.389)	
Endocrine therapy		0.0003		0.0008		0.0052		0.0043
Not done	ref		ref		ref		ref	
Done	1.164 (1.071–1.264)		1.153 (1.060–1.253)		1.167 (1.047–1.300)		1.171 (1.051–1.305)	
Radiation		0.0148		0.0517		0.5759		0.602
Not done	ref		ref		ref		ref	
Done	1.121 (1.023–1.230)		1.097 (0.999–1.203)		1.036 (0.916–1.172)		1.033 (0.913–1.170)	

CCI, Charlson Comorbidity Index.

## Data Availability

All data generated or analyzed during this study are included in this research article and Supplementary Information files. However, the original data are prohibited from being exported outside, due to NHI policy.

## Supplementary Materials

The following supporting information can be downloaded at https://www.mdpi.com/article/10.3390/cancers18030397/s1, Supplementary Figure S1. Kaplan–Meier analysis of the incidence of sleep disorder according to endocrine regimen in patients with breast cancer not receiving chemotherapy. (median follow-up, 9.37 ± 2.42 years). (a) cumulative incidence of sleep disorder before matching (b) cumulative incidence of sleep disorder after matching. Supplementary Figure S2. Annual hazard rate of the incidence of sleep disorder in patients with breast cancer patients. (a) before matching, annual hazard rate of the sleep disorder according to endocrine treatment in patients with breast cancer not receiving chemotherapy, (b) after matching, annual hazard rate of the sleep disorder according to endocrine treatment in patients with breast cancer not receiving chemotherapy, (c) before matching, annual hazard rate of the sleep disorder according to taxane use in patients with breast cancer receiving chemotherapy, (d) after matching, annual hazard rate of the sleep disorder according to taxane use in patients with breast cancer receiving chemotherapy. Supplementary Table S1. Surgical codes for breast and axillary surgery. Supplementary Table S2. Diagnostic codes for mental illness. Supplementary Table S3. Drug prescription codes. Supplementary Table S4. Charlson Comorbidity Index. Supplementary Table S5. Comparison of clinical characteristics according to endocrine regimen in breast cancer patients not receiving chemotherapy. Supplementary Table S6. Risk of sleep disorder in breast cancer patients according to endocrine regimen from analyses using the Cox proportional hazard models (Definition 2).
