# Supplementary material for: Association Between Therapeutic Interventions and Sleep Disorders in Patients with Breast Cancer: A National Population-Based Cohort Study
Source: Cancers (Basel). 2026 Jan 27;18(3):397. doi: 10.3390/cancers18030397 (PMC12896395; doi:10.3390/cancers18030397)
Supplement: Supplementary file 1 [file cancers-18-00397-s001.zip › cancers-3973502-supplementary.pdf]

Supplementary material

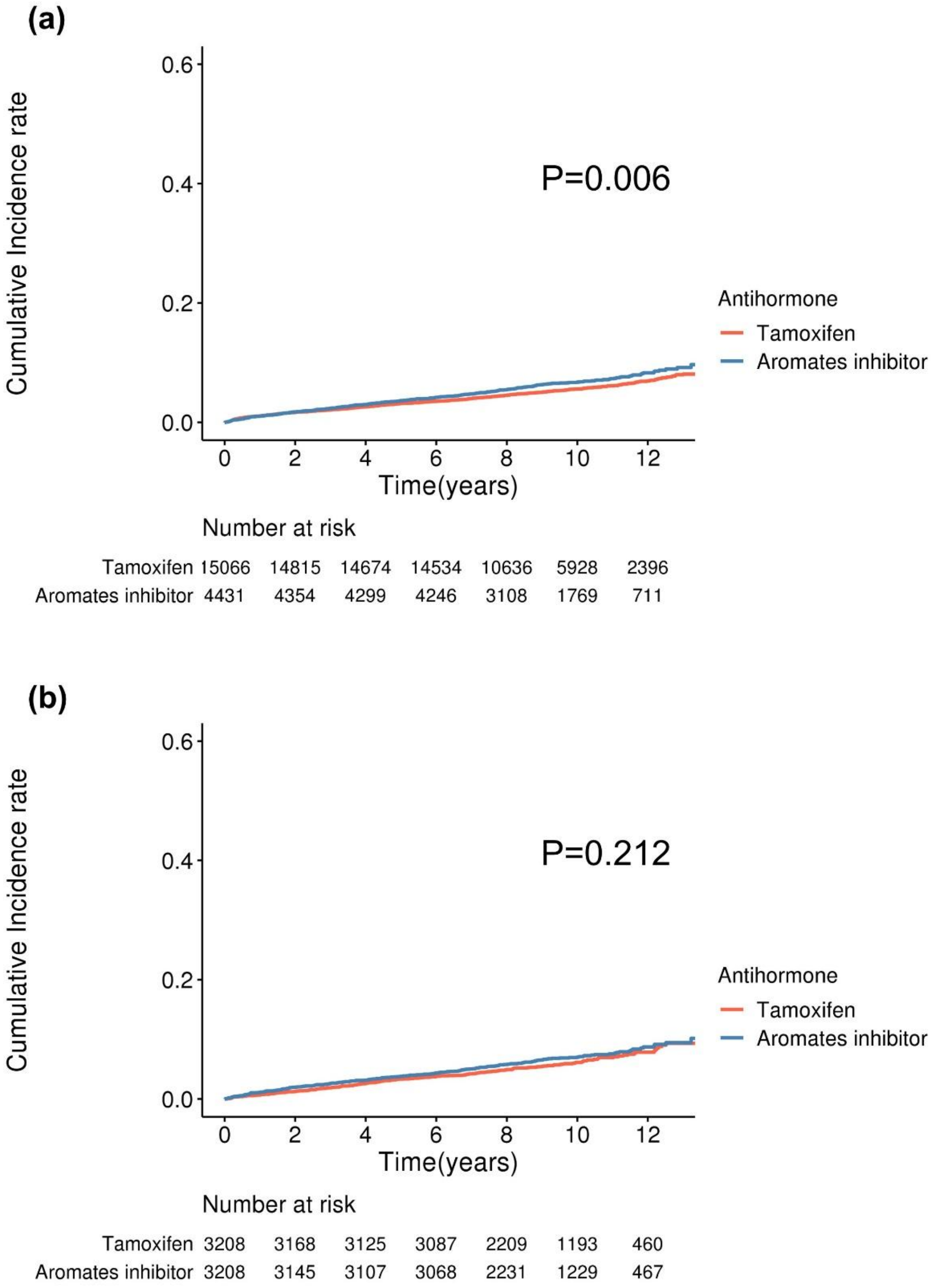

Supplementary Figure S1. Kaplan–Meier analysis of the incidence of sleep disorder according to endocrine regimen in patients with breast cancer not receiving chemotherapy. (median follow-up, 9.37 ± 2.42 years). (**a**) cumulative incidence of sleep disorder before matching (**b**) cumulative incidence of sleep disorder after matching.

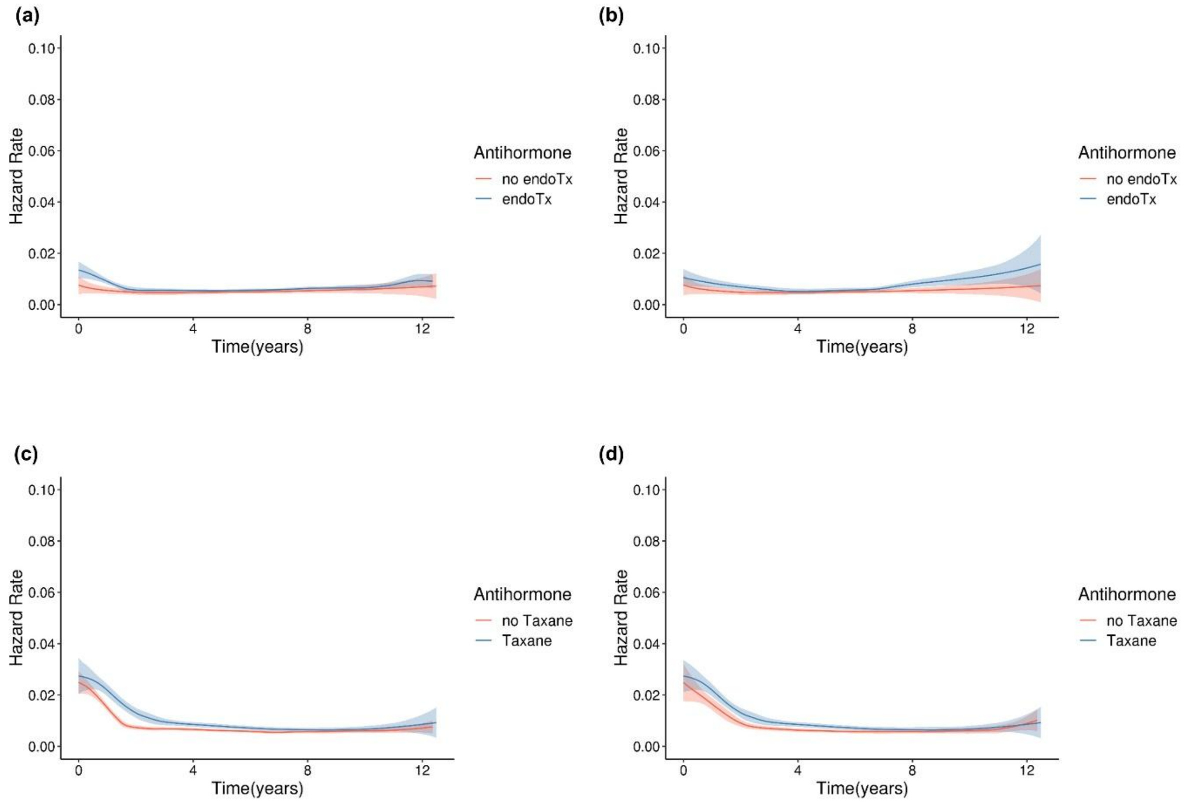

Supplementary Figure S2. Annual hazard rate of the incidence of sleep disorder in patients with breast cancer patients. (**a**) before matching, annual hazard rate of the sleep disorder according to endocrine treatment in patients with breast cancer not receiving chemotherapy, (**b**) after matching, annual hazard rate of the sleep disorder according to endocrine treatment in patients with breast cancer not receiving chemotherapy, (**c**) before matching, annual hazard rate of the sleep disorder according to taxane use in patients with breast cancer receiving chemotherapy, (**d**) after matching, annual hazard rate of the sleep disorder according to taxane use in patients with breast cancer receiving chemotherapy.

Supplementary Table S1. Surgical codes for breast and axillary surgery.

Behavior Code	ICD-10 Code
N7131	Simple mastectomy
N7132	Subcutaneous mastectomy
N7133	Mastectomy (partial)
N7133	Wide excision
N7134	Wide excision of axillary breast
N7135	Radical mastectomy including modified radical mastectomy and radical wide excision with axillary surgery
N7136	Wide excision with axillary surgery
N7137	Wide excision without axillary surgery
N7138	Total mastectomy with axillary surgery
N7139	Total mastectomy without axillary surgery

Supplementary Table S2. Diagnostic codes for mental illness.

Classification	Diagnosis	ICD-10 Code
**Anxiety disorder**	Agoraphobia	F40.0
	Social phobias	F40.1
	Specific (isolated) phobias	F40.2
	Other phobic anxiety disorders	F40.8
	Phobic anxiety disorder, unspecified	F40.9
	Panic disorder [episodic paroxysmal anxiety]	F41.0
	Generalized anxiety disorder	F41.1
	Mixed anxiety and depressive disorder	F41.2
	Anxiety depression(mild or not persistent)	F41.2
	Other mixed anxiety disorders	F41.3
	Other specified anxiety disorders	F41.8
	Anxiety disorder, unspecified	F41.9
**Depression disorder**	Mild depressive episode	F32.0
	Moderate depressive episode	F32.1
	Severe depressive episode without psychotic symptoms	F32.2
	Severe depressive episode with psychotic symptoms	F32.3
	Other depressive episodes	F32.8
	Depressive episode, unspecified	F32.9
	Recurrent depressive disorder, current episode mild	F33.0
	Recurrent depressive disorder, current episode moderate	F33.1
	Recurrent depressive disorder, current episode severe without psychotic symptoms	F33.2
	Recurrent depressive disorder, current episode severe with psychotic symptoms	F33.3
	Recurrent depressive disorder, currently in remission	F33.4
	Other recurrent depressive disorders	F33.8
	Recurrent depressive disorder, unspecified	F33.9

	Dysthymia	F34.1
	Other persistent mood[affective] disorders	F34.8
	Persistent mood[affective] disorder, unspecified	F34.9
	Other single mood[affective] disorders	F38.0
	Other recurrent mood[affective] disorders	F38.1
	Other specified mood [affective] disorders	F38.8
	Unspecified mood[affective] disorder	F39
**Sleep disorder**	Nonorganic insomnia	F51.0
	Nonorganic hypersomnia	F51.1
	Nonorganic disorder of the sleep-wake schedule	F51.2
	Sleepwalking [somnambulism]	F51.3
	Sleep terrors [night terrors]	F51.4
	Nightmares	F51.5
	Other nonorganic sleep disorders	F51.8
	Nonorganic sleep disorder, unspecified	F51.9
	Disorders of initiating and maintaining sleep [insomnias]	G47.0
	Disorders of excessive somnolence [hypersomnias]	G47.1
	Disorders of the sleep-wake schedule	G47.2
	Obstructive sleep apnoea	G47.30
	Central sleep apnoea	G47.31
	Mixed sleep apnoea	G47.32
	Other sleep apnoea	G47.38
	Narcolepsy and cataplexy	G47.4
	Other sleep disorders	G47.8
	Personal history of unhealthy sleep-wake schedule	Z91.3

Supplementary Table S3. Drug prescription codes.

Drugs	*Codes
**Endocrine drug**	
**Tamoxifen**	234501ATB, 234502ATB
**Aromatase inhibitor**	
Letrozole	182201ATB
Anastrozole	109001ATB
Exemestane	358401ATB
**Chemotherapy drug**	
**Taxane**	148301BIJ, 148302BIJ, 148303BIJ, 148304BIJ, 148305BIJ, 148306BIJ, 148309BIJ, 148310BIJ, 148338BIJ, 148339BIJ, 148340BIJ, 148341BIJ, 148342BIJ, 148344BIJ, 148345BIJ, 148346BIJ, 148348BIJ, 148349BIJ, 148350BIJ, 148351BIJ
**Doxorubicin**	149401BIJ, 149402BIJ, 149403BIJ, 149404BIJ, 149405BIJ, 149406BIJ, 149430BIJ, 149431BIJ, 149432BIJ, 149433BIJ, 149434BIJ, 149435BIJ
**Cyclophosphamide**	139001ATB, 139003BIJ, 139004BIJ, 139005BIJ
**Fluorouracil**	161401BIJ, 161402BIJ, 161404BIJ, 161430BIJ, 161431BIJ, 161432BIJ
**Methotrexate**	192101ATB, 192102BIJ, 192103BIJ, 192104BIJ, 192105BIJ, 192107ATB, 192107BIJ, 192108BIJ, 192109BIJ, 192110BIJ, 192111BIJ, 192132BIJ, 192134BIJ, 192136BIJ, 192138BIJ, 192139BIJ, 192140BIJ, 192141BIJ, 192142BIJ, 192143BIJ, 192144BIJ
**Carboplatin**	123703BIJ, 123730BIJ, 123701BIJ, 123731BIJ, 123702BIJ, 123732BIJ, 123707BIJ, 123733BIJ, 123708BIJ, 123735BIJ, 123704BIJ, 123734BIJ, 123706BIJ, 123736BIJ
**Doxepin**	149204ATB, 451901CCM, 149203ATB
**Trazodone**	242903ATR, 242901ATB, 242902ATB, 242901ACH
**Eszopiclone**	680401ATB, 680402ATB, 680403ATB
**Zolpidem**	250502ATB, 250501ATB, 250501ATD
**Zolpidem CR**	250504ATR, 250503ATR

**Triazolam**	243501ATB, 243502ATB

* Drug claim codes at National Health Insurance Service

Supplementary Table S4. Charlson Comorbidity Index.

Comorbidity	ICD-10 Code	Updated Weight
Myocardial infarction	I21.x, I22.x, I25.2	0
Congestive heart failure	I09.9, I11.0, I13.0, I13.2, I25.5, I42.0, I42.5-I42.9, I43.x, I50.x, P29.0	2
Peripheral vascular disease	I70.x, I71.x, I73.1, I73.8, I73.9, I77.1, I79.0, I79.2, K55.1, K55.8, K55.9, Z95.8, Z95.9	0
Cerebrovascular disease	G45.x, G46.x, H34.0, I60.x, I69.x	0
Dementia	F00.x-F03.x, F05.1, G30.x, G31.1	2
Chronic pulmonary disease	I27.8, I27.9, J40.x-J47.x, J60.x-J67.x, J68.4, J70.1, J70.3	1
Rheumatologic disease	M05.x, M06.x, M31.5, M32.x, M34.x, M35.1, M35.3, M36.0	1
Peptic ulcer disease	K25.x-K28.x	0
Mild liver disease	B18.x, K70.0-K70.3, K70.9, K71.3-71.5, K71.7, K73.x, K74.x, K76.0, K76.2-K76.4, K76.8, K76.9, KZ94.4	2
Diabetes without chronic complication	E10.0, E10.1, E10.6, E10.8, E10.9, E11.0, E11.1, E11.6, E11.8, E11.9, E12.0, E12.1, E12.6, E12.8, E12.9, E13.0, E13.1, E13.6, E13.8, E13.9, E14.0, E14.1, E14.8, E14.9	2
Diabetes with chronic complication	E10.2-E10.5, E10.7, E11.2-E11.5, E11.7, E12.2-E12.5, E12.7, E13.2-E13.5, E13.7, E14.2-E14.5, E14.7	1
Hemiplegia or paraplegia	G04.1, G11.4, G80.1, G80.2, G81.x, G82.x, G83.0-G83.4, G83.9	2
Renal disease	I12.0, I13.1, N03.2-N03.7, N05.2-N05.7, N18.x, N19.x, N25.0, Z49.0-Z49..2, Z94.0, Z99.2	1

Any malignancy including leukemia and lymphoma	There are no eligible patients in this study	2
Moderate or severe liver disease	I185.0, I85.9, I86.4, I98.2, K70.4, K71.1, K72.1, K72.9, K76.5, K76.6, K76.7	4
Metastatic solid tumor	There are no eligible patients in this study	6
Acquired immune deficiency syndrome/human immunedeficiency virus	B20.x-B22.x, B24.x	4

Supplementary Table S5. Comparison of clinical characteristics according to endocrine regimen in breast cancer patients not receiving chemotherapy.

	**Before Matching**			**After Matching**		
	**Tamoxifen, n = 15,066 (%)**	**Aromatase Inhibitor, n = 4,431 (%)**	***p* Value**	**Tamoxifen, n = 3,208 (%)**	**Aromatase Inhibitor, n = 3,208 (%)**	***p* Value**
**Age (year)**			<0.001			0.995
**20-29**	199 (1.32)	40 (0.09)		4 (0.12)	4 (0.12)	
**30-39**	2318(15.39)	25(0.56)		25(0.78)	25(0.78)	
**40-49**	8710(57.81)	188(4.24)		188(5.86)	188(5.86)	
**50-59**	2733(18.14)	2006(45.27)		1934(60.29)	1934(60.29)	
**60-69**	732(4.86)	1389(31.35)		708(22.07)	710(22.13)	
**70-79**	320(2.12)	720(16.25)		301(9.38)	300(9.35)	
**80-**	54 (0.36)	99 (2.2)		48 (1.5)	47(1.47)	
**Gender**			<0.001			>0.9999

**Male**	91(0.60)	0(0.00)		0(0.00)	0(0.00)	
**Female**	14975(99.40)	4431(100.00)		3208(100.00)	3208(100.00)	
**CCI (Weight number, mean±SD)**	3.251±1.644	4.047±2.179	<.0001	3.73±1.856	3.738±1.872	0.8618
**Radiation**			0.9242			0.8882
**Not done**	4178(27.73)	1232(27.80)		865(26.96)	870(27.12)	
**Done**	10888(72.27)	3199(72.20)		2343(73.04)	2338(72.88)	
**Sleep disorder (Definition 1)**			0.0132			0.1655
**No**	13842(91.88)	4019(90.70)		2931(91.37)	2899(90.37)	
**Yes**	1224(8.12)	412(9.30)		277(8.63)	309(9.63)	
**Sleep disorder (Definition 2)**			0.0049			0.1894
**No**	14229(94.44)	4135(93.32)		3011(93.86)	2985(93.05)	
**Yes**	837(5.56)	296(6.68)		197(6.14)	223(6.95)	
**Anxiety disorder (Definition 1)**			<.0001			0.7345
**No**	13491(89.55)	3864(87.20)		2810(87.59)	2801(87.31)	

**Yes**	1575(10.45)	567(12.80)		398(12.41)	407(12.69)	
**Anxiety disorder (Definition 2)**			<.0001			0.9014
**No**	13838(91.85)	3980(89.82)		2880(89.78)	2883(89.87)	
**Yes**	1228(8.15)	451(10.18)		328(10.22)	325(10.13)	
**Depression disorder (Definition 1)**			0.2614			0.3207
**No**	13479(89.47)	3938(88.87)		2839(88.50)	2864(89.28)	
**Yes**	1587(10.53)	493(11.13)		369(11.50)	344(10.72)	
**Depression disorder (Definition 2)**			0.2112			0.701
**No**	13707(90.98)	4004(90.36)		2900(90.40)	2909(90.68)	
**Yes**	1359(9.02)	427(9.64)		308(9.60)	299(9.32)	

CCI, Charlson Comorbidity index; SD, standard deviation. * Definition 1: If the diagnosis code is confirmed on the claim, it is operationally defined as having been diagnosed with the disease. # Definition 2: If both the diagnosis code and medication for disease are identified on the claim, the patient is operationally defined as having been diagnosed with the disease.

Supplementary Table S6. Risk of sleep disorder in breast cancer patients according to endocrine regimen from analyses using the Cox proportional hazard models (Definition 2).

	**Before Matching**		**Before Matching**		**After Matching**		**After Matching**	
	**Univariate Analysis**		**Multivariate Analysis**		**Univariate Analysis**		**Multivariate Analysis**	
	HR (95% CIs)	***p* Value**	**HR (95% CIs)**	***p* Value**	**HR (95% CIs)**	***p* Value**	**HR (95% CIs)**	***p* Value**
**Age (per 10-year)**	1.092 (1.037-1.149)	0.001	1.076 (1.009-1.149)	0.026	0.997(0.889-1.118)	0.9536	1.019(0.903-1.150)	0.7597
**Gender**		0.4948		0.5135				
**Male**	ref		ref		N/A		N/A	
**Female**	1.407(0.528-3.754)		1.390(0.517-3.735)					
**CCI**	1.035(1.005-1.066)	0.0212	1.024(0.993-1.056)	0.1294	0.995(0.946-1.046)	0.8363	0.996(0.946-1.048)	0.8679
**Endocrine therapy**		0.006		0.5003		0.2126		0.2065
**Tamoxifen**	ref		ref		ref		ref	
**Aromatase inhibitor**	1.204(1.055-1.375)		1.058(0.898-1.246)		1.130(0.933-1.368)		1.132(0.934-1.371)	
**Radiation**		0.0942		0.0499		0.2184		0.2055
**Not done**	ref		ref		ref		ref	
**Done**	1.121(0.981-1.280)		1.145(1.000-1.310)		1.149(0.921-1.433)		1.159(0.922-1.458)	

CCI, Charlson Comorbidity index
